# *Aegle marmelos* Leaf Extract Phytochemical Analysis, Cytotoxicity, In Vitro Antioxidant and Antidiabetic Activities

**DOI:** 10.3390/plants10122573

**Published:** 2021-11-25

**Authors:** Wasim Ahmad, Mohd Amir, Adil Ahmad, Abuzer Ali, Amena Ali, Shadma Wahab, Harshita Abul Barkat, Mohammad Azam Ansari, Mohammad Sarafroz, Ayaz Ahmad, Md. Abul Barkat, Prawez Alam

**Affiliations:** 1 Department of Pharmacy, Mohammad Al-Mana College for Medical Sciences, Dammam 34222, Saudi Arabia; ayaza@machs.edu.sa; 2 Department of Natural Products and Alternative Medicine, College of Clinical Pharmacy, Imam Abdul Rahman Bin Faisal University, Dammam 31441, Saudi Arabia; matahmad@iau.edu.sa; 3 Department of Pharmacognosy and Phytochemistry, School of Pharmaceutical Education and Research, Jamia Hamdard, New Delhi 110062, India; adilyaqoob08@gmail.com; 4 Department of Pharmacognosy, College of Pharmacy, Taif University, P.O. Box 11099, Taif 21944, Saudi Arabia; abuali@tu.edu.sa; 5 Department of Pharmaceutical Chemistry, College of Pharmacy, Taif University, P.O. Box 11099, Taif 21944, Saudi Arabia; amrathore@tu.edu.sa; 6 Department of Pharmacognosy, College of Pharmacy, King Khalid University, Abha 61421, Saudi Arabia; sabdulwahab@kku.edu.sa; 7 Department of Pharmaceutics, College of Pharmacy, University of Hafr Al-Batin, Hafr Al-Batin 39524, Saudi Arabia; harshiab001@gmail.com (H.A.B.); abulbarkat05@gmail.com (M.A.B.); 8 Department of Epidemic Disease Research, Institute for Research and Medical Consultations (IRMC), Imam Abdulrahman Bin Faisal University, P.O. Box 1982, Dammam 31441, Saudi Arabia; maansari@iau.edu.sa; 9 Department of Pharmaceutical Chemistry, College of Clinical Pharmacy, Imam Abdul Rahman Bin Faisal University, Dammam 31441, Saudi Arabia; mskausar@iau.edu.sa; 10 Department of Pharmacognosy, College of Pharmacy, Prince Sattam Bin Abdulaziz University, P.O. Box 173, Al-Kharj 11942, Saudi Arabia; prawez_pharma@yahoo.com

**Keywords:** *Aegle marmelos*, HepG_2_ cells, GC/MS, HPLC, diabetes, cytotoxicity, antioxidant

## Abstract

For many years, *Aegle marmelos* (*A. marmelos*) has been used medicinally and as a dietary supplement. Despite this, there are minimal research data on *A. marmelos* phytochemical properties and pharmacological effects. This study aimed to explore the phytoconstituents, cytotoxicity, glucose uptake, and antioxidant and antidiabetic potential of an alcoholic extract of *A. marmelos* leaf. The cytotoxicity of *A. marmelos* in HepG2 cells was tested in vitro, and the results revealed that it has strong cytocompatibility and cytoprotective properties. The extract’s antioxidant activities were investigated using 2,2-diphenyl-1-picrylhydrazyl (DPPH) and ferric reducing antioxidant power (FRAP) methods. Antioxidant potential was shown to be quite impressive. The enzymes α-amylase and α-glycosidase were found to be substantially inhibited by *A. marmelos*, with IC_50_ values of 46.21 and 42.07 mg/mL, respectively. In HepG2 cells, *A. marmelos* significantly reduced ROS levels that were elevated due to high glucose and enhanced glucose consumption (*p* < 0.05). These activities might be due to the enrichment of bioactive phytoconstituents analyzed chromatographically using GC/MS and HPLC. The findings of this study show that *A. marmelos* could be an effective restorative therapy for diabetes and related diseases.

## 1. Introduction

Diabetes is a chronic metabolic disease that poses a threat to both human health and the global economy [1]. According to an IDF estimate, 463 million people worldwide had diabetes in 2019 [2]. Diabetes has a complicated etiology that includes various features such as hyperglycemia, oxidative stress, and inflammation, leading to long-term damage and consequences [3]. Oxidative stress (OS), which results from chronic hyperglycemia, has emerged as a critical and common event in diabetes and cardiovascular disease development [4]. According to biomedical research, several harmful pathogeneses, such as diabetes mellitus, cardiometabolic illnesses, cancer, and brain-related disorders, are significantly associated with hyperglycemia and unstable free radicals in the biosystem. In people with diabetes, an imbalance between antioxidant enzymes and reactive oxygen species (ROS) convinces the OS to cause cell mutilation [5]. This activity can be decreased by taking antioxidant-rich dietary supplements, which neutralize or scavenge the oxidant. Despite the availability of current antidiabetic medications, diabetes treatment continues to be a challenge due to its limited therapeutic efficacy and adverse effects such as hypoglycemia, weight gain, gastrointestinal problems, insulin resistance, and cholestatic liver injury with long-term use [6,7]. As a result, it is critical to conduct a study of dietary supplements with antihyperglycemic properties. Such a study will likely play an essential role in developing a successful treatment for the management of diabetes and its complications.

Traditional medicine has gained popularity in recent decades due to the “back-to-nature” tendency in medicine selection. Many countries throughout the world are using traditional medicine derived from diverse medicinal plants to treat various diseases and conditions [8]. Nutritional supplements and nutraceutical traditional medicine are used by nearly 80% of the world’s population according to a WHO report, mainly in developing countries because of their extraordinary pharmacological potential and low toxicity, as well as the rarity of side effects. To date, many pharmaceutical substances have been discovered through the practice of traditional medicine; this may be a good domain to begin in the quest for new therapeutics [9,10]. Yet, the therapeutic-drug-making potential of plants as raw materials has largely been ignored [11].

*Aegle marmelos* is considered a food supplement in India and is included in the *Ayurvedic Pharmacopeia of India* due to their curative potential for conditions such as diabetes and related disorders. Although medicinal plants are revered and used in various systems of medicine such as Ayurveda, Unani, Chinese, and Tibetan medicine for therapeutic purposes, the majority of traditional medicines has not been experimentally evaluated or validated for their pharmacological efficacy [12]. Therefore, detailed and extended phytochemical profiling is necessary to establish the quality control of alcoholic extract of *A. marmelos*. GC/MS and HPLC are increasingly being utilized to characterize the structure of complicated matrices and have established themselves as the techniques of choice for phytochemical identification. In addition, no research on *A. marmelos’* anti-inflammatory, antioxidant, cytoprotective, and glucose-utilizing properties has been published to date. Hence, this study aimed to chromatographically explore its phytoconstituents. Furthermore, we investigated the toxicological, antioxidant, and pharmacological potential of *A. marmelos*.

## 2. Results

### 2.1. Chemical Profiling of A. marmelos Extract Using GC/MS

**Figure 1:**
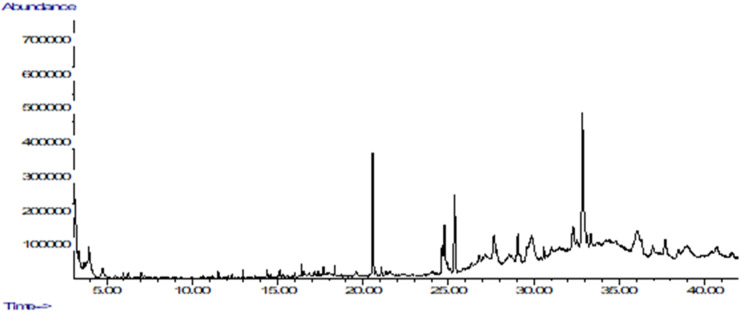
GC/MS total ion chromatogram of *A. marmelos*.

GC/MS profiling identified a total of 51 compounds. A list of the identified phytoconstituents is presented in Table 1. The typical chromatogram of the GC/MS analysis is depicted in Figure 1. The total ion chromatogram (TIC) peaks were matched with the phytoconstituents in the NIST GC/MS library.

### 2.2. Identification of Gallic Acid and Rutin Using HPLC

**Figure 2:**
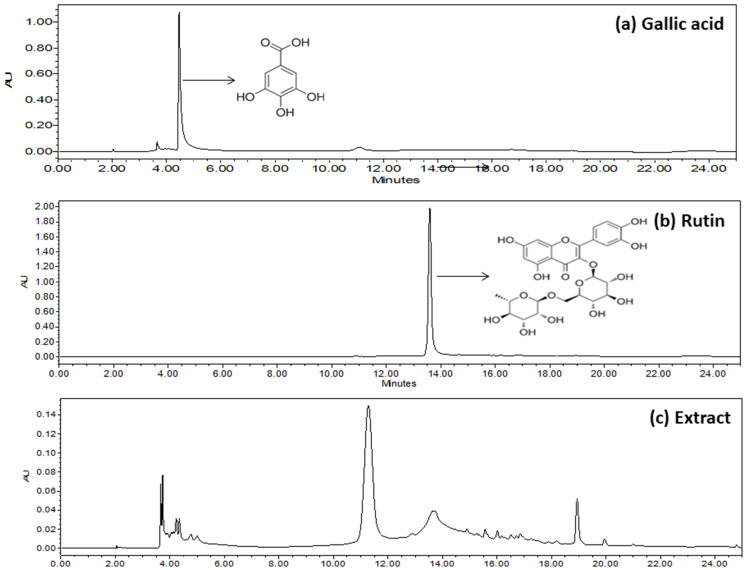
Typical HPLC chromatograms of (**a**) gallic acid standards, (**b**) rutin standards, and (**c**) *A. marmelos*.

An HPLC analysis of *A. marmelos* extracts established the presence of gallic acid (RT, 4.879) and rutin (RT, 13.567). The corresponding RT of the *A. marmelos* extract was matched with the standard phytoconstituents gallic acid and rutin (Figure 2).

### 2.3. Total Phenol and Flavonoid Content

The total phenolic and flavonoid content of the plant extract was estimated from the standard calibration curve of gallic acid (r^2^ = 0.9929) and rutin (r^2^ = 0.9946). The total phenolic and flavonoid content, equivalent per gram of extract to an aqueous extract of *A. marmelos*, was estimated to be 31.38 and 16.36 mg of gallic acid and rutin, respectively.

### 2.4. DPPH Antioxidant Activity

**Figure 3:**
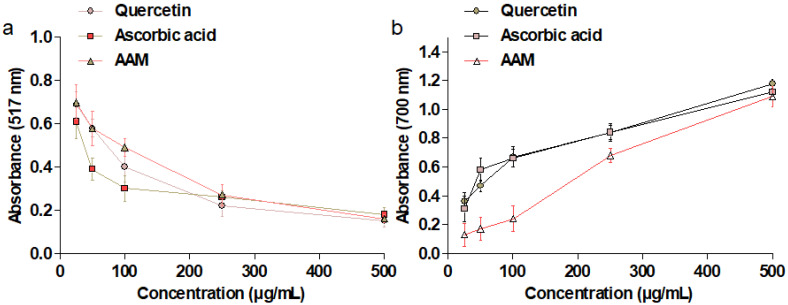
Antioxidant potential of *A. marmelos*: (**a**) DPPH radical scavenging activity and (**b**) FRAP assay.

The antioxidant (radical scavenging activity) potential of *A. marmelos* was assessed against DPPH radicals. The obtained results are presented in Figure 3a. The results showed significant inhibitory potential in graded dose–response on a DPPH free radical at the different tested concentrations (25–500 µg/mL). Initially, the reference compounds such as quercetin and ascorbic acid showed better inhibitory activity compared to the alcoholic extract of *A. marmelos* (AAM), but at higher concentrations, AAM showed similar antioxidant effects to the reference compounds.

### 2.5. Reducing Power of A. marmelos

In the present study, the ferric reducing power of *A. marmelos* showed excellent inhibition potential, but no better than the reference compounds (Figure 3b). In the FRAP assay, a subsequent color change from yellow to green was observed after reducing Fe^3+^ to Fe^2+^ [13]. The obtained results revealed that the extracts have polyphenols containing hydroxyl groups in their skeletons.

### 2.6. In Vitro α-Amylase and α-Glucosidase Activity

In the current investigation, *A. marmelos* was assessed for its possible α-amylase and α-glucosidase inhibitory action using acarbose as the standard. The IC_50_ values of *A. marmelos* compared to α-amylase (IC_50_ 123.65 µg/mL) and α-glucosidase were found to be IC_50_ 123.65 µg/mL and IC_50_ 141.56 µg/mL, respectively. The potential inhibitory activity of *A. marmelos* (25–500 µg/mL) was observed to be concentration-dependent. The results revealed that *A. marmelos* was more effective against α-amylase and α-glucosidase than against acarbose (IC_50_ 153.16 µg/mL).

### 2.7. Cytotoxicity and Cytoprotective Assay

**Figure 4:**
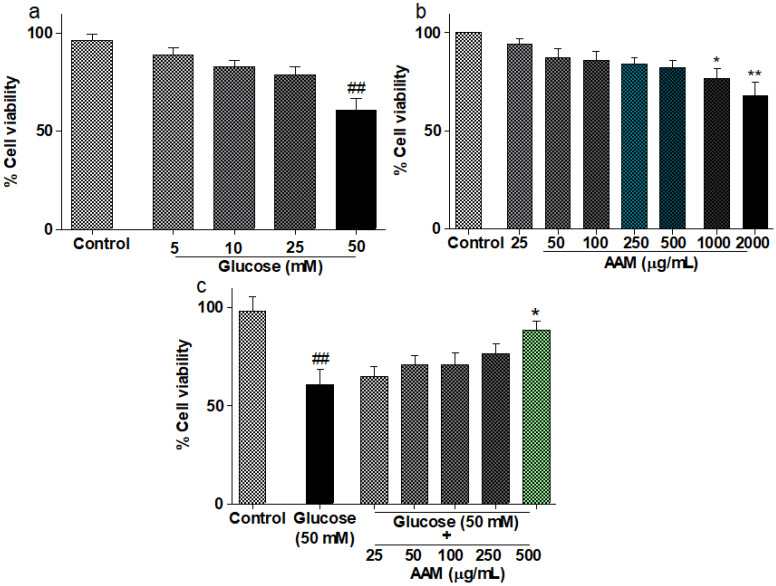
The effects of (**a**) glucose and (**b**) *A. marmelos* on the HepG_2_ cells viability. (**c**) Cytoprotective effect of *A. marmelos* compared to glucose-induced hyperglycemia in HepG_2_ cells for 24 h. Values are presented as mean ± SD (n = 3). */^#^, statistically significant versus glucose/control, respectively (* *p* < 0.05, **/^##^ *p* < 0.01).

The results demonstrated that 50 mM glucose caused significant (*p* < 0.05) toxicity to HepG2 cells, but lower doses were not toxic (Figure 4b). Additionally, *A. marmelos* exhibited substantial (*p* < 0.05) cytotoxicity at higher concentrations (1000–2000 μg/mL) compared to the standard control (Figure 4b). *A. marmelos*, on the other hand, was non-toxic at concentrations up to 500 μg/mL and maintained cell viability at 82.33% throughout the study. Interestingly, incubation with 50 mM glucose and *A. marmelos* (500 μg/mL) considerably increased cell viability (*p* < 0.05; Figure 4c).

### 2.8. Glucose Utilization Assay

**Figure 5:**
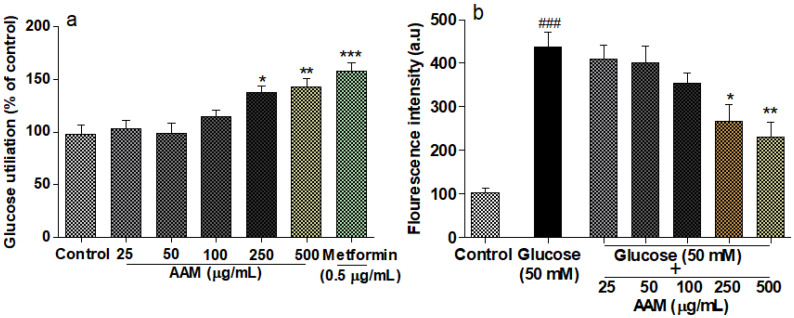
The (**a**) glucose utilization and (**b**) protective effect of *A. marmelos* on glucose induced ROS generation in HepG_2_ cells by DCF-DA for 24 h. Values are presented as mean ± SD (n = 3). */^#^, statistically significant versus glucose/control, respectively (* *p* < 0.05, ** *p* < 0.01, ***/^###^ *p* < 0.001).

The observation of glucose utilization in the HepG2 cells of *A. marmelos* (25–500 µg/mL) and metformin is shown in Figure 5a. In a dose-dependent manner, *A. marmelos* significantly (*p* < 0.05) improved glucose utilization. However, *A. marmelos* increased glucose utilization in HepG2 cells by 143% ± 6.80% at higher concentrations, but this utilization was lesser than that of the standard drug metformin (158% ± 5.56%).

### 2.9. Cellular Antioxidant Activity

Incubating HepG2 cells with 50 mM glucose for 24 h (*p* < 0.05) significantly enhanced cellular ROS levels beyond what we observed in the control. *A. marmelos* treatment at doses above 100 µg/mL (*p <* 0.05) significantly reduced the level of ROS (Figure 5b). The present study employed 2,7-dichlorofluorescein diacetate (DCF-DA) as a free radical species generator in the HepG2 cells. Damage to hepatocytes is widespread because liver cells are diligently involved in detoxifying several toxic substances.

## 3. Discussion

The GC/MS study demonstrated that *A. marmelos* has a diverse array of bioactive phytoconstituents, most of which are responsible for the plant’s pharmacological activity. Thus, GC/MS is a vital chromatographic technique frequently employed to identify phytoconstituents in plant extracts, fractions, and raw materials. The present investigation found the primary bioactive phytoconstituents, including 13-docosenoic acid (14.58), octadecanoic acid (4.87), oleic acid (3.52), retinoic acid (3.33), linolenic acid (2.72), myristic acid (0.70), gamma-tocopheryl (0.30), p-cymene (0.22), and vitamin-E (0.21). These compounds exhibit remarkable antioxidant, anti-inflammatory, and antidiabetic activity [14]. Among all identified phytoconstituents, oleic acid and p-cymene exhibit insulin sensitivity and improve β-cell survival [15,16]. Belurey et al. reported that linolenic acid significantly reduced the risk of diabetes and enhanced insulin sensitivity [17]. It was also reported that linolenic acid possesses antioxidant, immunomodulatory, and hypolipidemic effects [18]. Vitamin-E exerts antioxidant activity through a free radical scavenging mechanism. It also delays the occurrence of diabetes progression [16]. The cited study reported that myristic acid ameliorates the risk of diabetes progression and/or development in vivo by improving insulin sensitivity [19]. In addition, the most prevalent phytoconstituents in medicinal plants are gallic acid and rutin. A qualitative examination of these phytoconstituents is becoming increasingly relevant for quality control purposes, as well as to determine their pharmacological potential.

Polyphenols such as gallic acid and rutin, found abundantly in plants, may beneficially influence hyperglycemia by inhibiting digestive enzymes (amylase and glucosidase), stimulating insulin secretion from the β cells of the pancreas, and decreasing blood glucose levels. Furthermore, polyphenols protected the β cells and their integrity by modulating hyperglycemia and OS [20,21]. The discovery of gallic acid and rutin in *A. marmelos* bolstered the role of flavonoid and phenolic compounds in antioxidant and antidiabetic activity. The total phenolic and flavonoid content results revealed that *A. marmelos* is high in polyphenols, which are primarily responsible for its antioxidant and antidiabetic properties. The antioxidant potential of *A. marmelos* was found to be almost similar to reference compounds such as quercetin and ascorbic acid. The reduction capacity suggests that *A. marmelos* is rich in sources of bioactive metabolites, which have electron donor compounds and can therefore reduce any oxidized intermediates in the reaction medium.

Apart from scavenging the free radicals, it has been reported that the phenolic and flavonoids powerfully protect the generation of free radicals in the living organism and have an additive effect on the endogenous scavenging compounds [22,23]. Based on the findings, we hypothesize that our tested extract possesses excellent antioxidant activity. Moreover, radical scavenging activities are paramount to arresting the destructive role of free radicals in various diseases, including diabetes.

The degree of color change indicated the antioxidant capacity of the extract in the ferric-reducing power assay of *A. marmelos*. Thus, we anticipated a direct and significant relationship between the antioxidant and reducing capacity of the bioactive phytoconstituents found in plant extracts [17]. The findings of this study revealed that *A. marmelos* possesses an extraordinary ability to donate an electron to reactive free radicals, transitioning them to more stable non-reactive species, reducing oxidized intermediates, and acting as primary antioxidants. Surprisingly, the extract exhibited an exceptional capacity for total antioxidants. Our findings are consistent with prior research and imply that the antioxidant capacity of the plant is a result of its phytoconstituents, particularly polyphenols.

Increasing evidence from experimental and clinical studies suggests that inhibition of α-amylase and α-glucosidase by phytoconstituents ameliorates postprandial hyperglycemia and hyperlipidemia by interfering with the activity of α-amylase and α-glucosidase, hence preventing the onset of diabetes. According to Villa-Rodriguez et al., plant extracts are effective remedial therapeutic agents that diminish digestion and carbohydrates [24]. Yang et al. also described that excessive expression of the enzyme amylase, insulin insufficiency, or blood glucose level might increase and turn to hyperglycemia [25]. Hence, it is assumed that *A. marmelos* may be used as remedial therapy to restore a higher level of blood glucose to normal, which can potentially suppress the progression of diabetes.

Conversely, our observation showed that *A. marmelos* protects hepatocytes against high-glucose-induced hyperglycemia and OS in HepG2 cells, indicating a more significant safety margin of bioactive phytoconstituents available in the samples. Kong et al. reported that the cellular protection potential of the phytoconstituents is mainly dependent on their concentration, bioaccumulation, and synergic effect among the phytoconstituents [26].

A previously cited study revealed that bioactive compounds such as polyphenols could reduce the glucose release from hepatic cells and stimulate glucose uptake by regulating the cellular signaling pathway [19,27]. Therefore, based on our experimental findings, we can assume that the glucose uptake observed in hepatic cells might be due to the presence of polyphenols in *A. marmelos*. We hypothesize that *A. marmelos* could activate the insulin signaling cascade, resulting in stimulation of the glucose transporter (GLUT 2) that promotes the transport of glucose into the cell [28].

In addition, OS is a pathological condition in which the system generates excessive ROS, overwhelming the antioxidant’s ability to remove ROS from the system [29]. ROS can damage biomolecules, including carbohydrates, DNA, proteins, and lipids, ultimately resulting in cell apoptosis. Even the long-term existence of OS eventually ushers in intensifying loss of cell integrity and functions that lead to several complications such as diabetes, cardiovascular disorders, ageing, cancer, and atherosclerosis. ROS has been implicated in several cellular pathways such cell proliferation, c-Jun amino-terminal kinase, mitogen-activated protein kinase signaling, and cell apoptosis [30]. ROS not only harms cellular function and integrity, but it is also crucial for regulating cell signaling, and the level of ROS produced may influence this. Therefore, inhibiting the excess generation of ROS is crucial to providing appropriate targets to evolve innovatory and efficacious therapeutic strategies. Moreover, inhibiting the excess generation of ROS or the maintenance of hyperglycemia restores metabolic and vascular imbalances and prevents the onset and progression of diabetic complications [5]. Interestingly, the ROS inhibitory effect of *A. marmelos* was suggested to indicate that *A. marmelos* has the potency to ameliorate ROS-produced OS and hepatic cell damage and maintain cell integrity and functions. Overall, AAM impressively possesses cytocompatibility, antioxidant, and antidiabetic potential, as well as improved cellular glucose uptake, which might be due to the presence of phytoconstituents in the extract.

## 4. Materials and Methods

### 4.1. Chemicals and Plant Material

The reagents and chemicals used in the experiments were purchased from Amplicon Biotech, New Delhi, India and Himedia Laboratories, Ltd. Solvents used for extraction (AR grade), GC/MS, and HPLC (analytical grade) were purchased from Merck (New Delhi, India). The fresh leaves of *A. marmelos* were obtained locally, and taxonomic identification was made (Voucher specimen number; IU/2020/*Aegle marmelos*).

### 4.2. Preparation of Alcoholic Extract of A. marmelos

The leaves of *A. marmelos* were collected, shade-dried, and pulverized into coarse powder using a digital grinder. Next, 50 g of *A. marmelos* leaf powder was accurately weighed and then extracted with 400 mL of ethanol and transferred to a flask in a water bath at 40 °C for 3 h, stirring occasionally. It was filtered through No. 1 Whatman filter paper and then washed with a fresh solvent to remove any remaining residue. The filtrate and washing were pooled and evaporated to dryness on rotavapor below 60 °C. The obtained residue was stored in well-closed containers for further studies. Additionally, the obtained alcoholic extract contained both polar phytoconstituents and nonpolar phytoconstituents.

### 4.3. GC/MS Analysis

A derivatization reagent (N-methyl-N-trifluoroacetamide with 1% trimethylchlorosilane) was used to derivatize the extract prior to GC/MS (Agilent 7890A) analysis, and the prepared sample was incubated for 2 h at room temperature with occasional vortexes. The diluted sample of 2.0 μL was injected (10:1 split ratio onto a 30 m × 0.25 mm × 0.25 μm HP-5MS column). Detailed instrumentation is given in Supplementary Table S1. The MS phytoconstituents were identified by matching the mass spectra of the analytes with the mass spectra of standards in NIST libraries and published data [31].

### 4.4. Identification of Gallic Acid and Rutin in A. marmelos Using HPLC

As per the reported methods with slight modification, HPLC with a variable wavelength (λmax) and a PDA detector was used to identify standard markers (gallic acid and rutin) in the samples [32]. The detailed instrumental condition of HPLC (Waters Alliance (e2695), Dover, NH, USA) is given in Table 2. Before using the mobile phase, it was ultrasonically degassed and filtered through a nylon membrane filter (0.22 µm). The (λmax) was set at 278 nm for gallic acid and rutin. The standard and sample were injected at a volume of 50 µL at a flow rate of 1.0 mL/min. The overall duration of the run was set to 25 min. Finally, the presence of gallic acid and rutin in the sample was confirmed by matching it with the retention time of the standard.

### 4.5. Estimation of Total Phenol and Flavonoid Content

A colorimetric assay was utilized to estimate the total phenolic and flavonoid content [33]. A volume of 5 µL of *A. marmelos* extract and 50 µL of 1 mM sodium carbonate was added, followed by the addition of Folin-Ciocalteu reagent (diluted 1:10 with deionized water) to bring the volume to 150 µL in 96-well plates. The plates were incubated at 40 °C for 30 min, and the absorbance at 765 nm was recorded. For flavonoid content, aliquots were prepared by mixing the test drug (*A. marmelos* extract) and aqueous AlCl_3_ (20% *w/v*). The absorptions were recorded at 425 nm after 10 min of incubation. All of the experiments were carried out in triplicate to ensure reproducibility. Gallic acid and rutin were used as reference compounds to plot the calibration curve to estimate phenol and flavonoid content.

### 4.6. In Vitro DPPH Assay

The extract of *A. marmelos* was assessed for its DPPH radical scavenging activities using the technique reported previously [34]. Different concentrations of *A. marmelos* extract (25–500 μg/mL) were added to 1 mM DPPH solution and then placed in a dark chamber at room temperature for 30 min. After, the absorbance of the mixture was recorded spectrophotometrically at 517 nm. Decreased absorbance illustrated the DPPH free radical scavenging potential. As a positive control, ascorbic acid and quercetin were utilized.

### 4.7. Ferric-Reducing Antioxidant Power (FRAP) Assay

Briefly, the reaction mixture included different concentrations of *A. marmelos* extract (25–250 μg/mL); 0.2M of 2.5 mL of phosphate buffer (pH 6.6); potassium ferricyanide (2.5 mL, 1% *w/v*). After a 20 min incubation at 50 °C, 2.5 mL of trichloroacetic acid (10%) and 0.5 mL of FeCl_3_ (0.1%) were added. At 700 nm, absorbance was recorded in comparison to blank samples (mixture without samples). Positive controls included ascorbic acid and quercetin. The reducing power of iron from (Fe^3+^) to Fe^2+^ was evaluated in triplicate [34].

### 4.8. Inhibitory Activity of α-Amylase and α-Glucosidase

Different concentrations of *A. marmelos* extract (25–500 µg/mL) were used to evaluate α-amylase and α-glucosidase inhibition potential [35]. Briefly, for the α-amylase assay, 1.0 mL of different concentrations of *A. marmelos* extract and 1.0 mL α-amylase were mixed by gentle shaking and incubated for 30 min at 37 °C; then, 1.0 mL of starch solution was added and the solution was incubated for 1 h at the same condition. Furthermore, 100 µL of supernatant was removed, and the inhibitory activity of *A. marmelos* extract was measured. For the α-glucosidase assay, 120 µL of different concentrations of *A. marmelos* extract and 20 µL of α-glucosidase were incubated for 15 min, and the reaction was carried out by adding 20 µL of 5 mM *p*-nitrophenyl-α-D-glucopyranoside substrate. The reaction was terminated by adding 80 µL potassium phosphate buffer, and absorbance was recorded at 405 nm. Acarbose was employed as a positive control. The below Equation (1) articulates the results:

(1) $$\%\;\mathrm{Inhibition}\;=\frac{A_{control}-A_{sample}}{A_{control}}$$

### 4.9. Cytotoxicity and Cytoprotective Assay

Dulbecco’s modified Eagle medium (DMEM), 10% fetal bovine serum, penicillin (100 U/mL), and streptomycin (100 μg/mL) were used to incubate (37 °C, 5% CO_2_) HepG2 cells (ATCC: HB-8065) in multiwell culture plates at a density of 1 × 10^5^ cells/mL. The MTT assay was performed to assess the potential of *A. marmelos* extract on the viability of the HepG2 cell line. Cells (1 × 10^5^/mL) were inoculated on 96-well plates and cultured with test samples (25–1000 μg/mL) for 24 h. A 20 μL MTT solution (5 mg/mL) was added, and the cells were again incubated for 4 h. The MTT solution was then discarded, and 150 μL DMSO (dimethylsulfoxide) was added to dissolve the formazan crystals [36]. The absorbance at 490 nm was determined using a multi-mode microplate reader.

### 4.10. Glucose Utilization Activity

The glucose utilization of the hepatocytes was estimated as per the standard methods with slight modification [37]. The HepG2 cells were cultured in the multi-well plate and incubated at room temperature (5% CO_2_) for three days. On day three, 10 µL of *A. marmelos* extract was added to each well at a concentration of 25, 50, 100, 250, or 500 µg/mL and incubated for 2 days. After 2 days, the media was discarded, a 25 µL incubation buffer (RPMI medium diluted with PBS, 0.1% BSA, and 8 mm glucose) was added, and the cells were incubated again for another 3 h. Subsequently, 10 µL of medium from the culture plate was transferred to a fresh multi-well plate, 200 µL of glucose oxidase reagent was added, and the absorbance was measured at 492 nm to determine the glucose concentration in the medium. The positive control was metformin (0.5 µg/mL), while the negative control comprised incubation buffer without samples. The percentage of glucose utilized was calculated using untreated controls.

### 4.11. Cellular Antioxidant Capacity

The levels of ROS in cells were determined using the approach reported before with minor modifications [38]. HepG2 cells (1 × 10^5^) were cultured in 6-well plates with or without glucose and incubated for 24 h at 37 °C with 5% CO_2_. Following that, selected wells were incubated for 48 h with varying concentrations of *A. marmelos* extract (25–500 μg/mL). After 48 h, 10 μM of the fluorescent probe DCF-DA was added and incubated for 30 min. After that, the cells were gently washed with PBS and, using a multi-mode fluorescence reader, the fluorescence intensity was recorded at 485 nm excitation and 535 nm emission, respectively.

### 4.12. Statistical Analysis

GraphPad Prism (version 5) was used to analyze the data using one-way ANOVA, followed by the Tukey’s multiple comparison tests, and the findings are reported as mean ± standard deviation (SD). A difference in mean values of *p* ≤ 0.05 was considered statistically significant.

## 5. Conclusions

The study provides pharmacological insight into the antioxidant, cytoprotective, and antidiabetic potential of *A. marmelos* extract with complete chemical profiling using GC/MS and HPLC. The findings revealed a high antioxidant potential, good cytocompatibility, and significant antidiabetic potential. The plant under investigation has the potential to be used alone or as an adjuvant therapy to avoid oxidative stress, diabetes, and its associated complications.

## Figures and Tables

**Table 1:** GC/MS profiling of *Aegle marmelos*.

S. No	Name of Metabolite	RT	%
1.	2-hexanone	3.643	0.38
2.	PENTAN-2-ONE	3.746	0.59
3.	Methyl-2-pentanone	3.841	0.60
4.	1-penten-3-ol	3.936	2.92
5.	Cyclopentanol	4.756	1.05
6.	p-mentha-1 (7),3-diene	6.250	0.36
7.	p-cymene	7.019	0.22
8.	8-hexadecenal	11.522	0.24
9.	(E)-1-(methoxymethoxy)-1-tetradecee-3-ol	12.972	0.26
10.	1-dodecanol	14.385	0.47
11.	Heneicosane	15.146	0.43
12.	Cyclooctasiloxane	16.420	0.57
13.	Tetradecamethylcycloheptasiloxane	16.508	0.42
14.	Dotriacontane	17.687	0.62
15.	Cyclononasiloxane	18.346	0.82
16.	Phthalic acid	19.613	0.56
17.	Hexadecanoic acid	20.572	7.23
18.	Cyclodecasiloxane	21.070	0.91
19.	Tetradecanoic acid (myristic acid)	21.326	0.70
20.	Methyl linolelaidate	24.628	1.86
21.	Oleic acid	24.767	3.52
22.	9,17-octadecadienal	24.906	0.99
23.	Octadecanoic acid	25.360	4.87
24.	Hentriacontane	26.334	2.26
25.	9,12,15-Octadecatrienoic acid, methyl ester (Linolenic acid, methyl ester)	26.788	2.72
26.	Octadecanoic acid (Stearic acid)	27.059	1.05
27.	Alpha-Neodene	27.147	0.80
28.	Sulfurous acid	27.191	0.82
29.	Phenol	27.660	4.09
30.	Tetracosamethyl-cyclododecasilaxane	27.835	2.42
31.	Oleyl alcohol	28.567	2.37
32.	2-propenoic acid, 3-(4-hydroxy-3-methoxyphenyl)-, methyl ester (cinnamic acid, 4-hydroxy-3-methoxy-, methyl ester)	28.692	0.87
33.	cis-13-eicosenoic acid	29.073	3.20
34.	Nonahexacontanoic acid	29.607	1.49
35.	Nonacosane	29.878	5.93
36.	Benzoic acid	31.035	1.36
37.	Vitamin-E	31.247	0.21
38.	Gamma-tocopheryl	31.504	0.30
39.	1-heptadec-1-ynyl-cyclopentanol	32.316	2.65
40.	7-pentadecyne	32.514	0.90
41.	13-docosenoic acid	32.851	14.58
42.	Docosanoic acid	33.334	2.53
43.	Z,Z-10,12-hexadecadien-1-ol acetat	34.220	0.71
44.	n-triacontane	36.080	5.67
45.	Tetracosamethyl-cyclododecasiloxane	36.299	2.53
46.	(+)-(9.beta.H)-labda-8(17),13(E)-diene-5-ol	36.973	0.84
47.	15-tetracosenoic acid	37.698	2.68
48.	Benchequiol	38.452	1.16
49.	Retinoic acid	38.979	3.33
50.	Cyclodecasiloxane	40.722	1.38
51.	Farnesyl acetone	41.601	0.82

**Table 2:** Optimized instrumental conditions for HPLC.

Parameter	Value	·
Mobile phase	Solvent A (0.5% formic acid in water) and solvent B (acetonitrile)	·
Stationary phase	C18 column (150 × 4.6 mm, particle size 5.0 µm, Phenomenex, Torrance, CA, USA)	·
Wavelength	278 nm	·
Solvent flow rate	1.0 mL/min	·
Gradient	Time (min)	Gradient ratio (Solvent A:Solvent B)
·	Initially	10:90
·	0–5	20:80
·	5–7	25:75
·	7–10	30:70
·	10–15	60:40
·	15–18	20:80
·	18–25	10:90

## Data Availability

The authors confirm that the data supporting the study’s findings are included in the article and its Supplementary Information.

## Supplementary Materials

The followingis available online at https://www.mdpi.com/article/10.3390/plants10122573/s1. Table S1: Detailed instrumentation of GC/MS profiling.
