# Supplementary material for: Aegle marmelos Leaf Extract Phytochemical Analysis, Cytotoxicity, In Vitro Antioxidant and Antidiabetic Activities
Source: Plants (Basel). 2021 Nov 25;10(12):2573. doi: 10.3390/plants10122573 (PMC8708738; doi:10.3390/plants10122573)
Supplement: Supplementary file 1 [file plants-10-02573-s001.zip › plants-1463841-supplementary.pdf]

Supplementary Table S1: Detailed instrumentation of GC/MS profiling.

**Instrument**	**Agilent**
Column	HP-5_{MS}
**Experimental condition**	
Inlet temperature	250ºC
Injection mode	Split (1:5)
Carrier gas	Helium
**Oven**	
Initial temperature	50ºC for 2 min
Ramp	10ºC/min. to 200ºC for 5 min, 5ºC/min. to 250ºC for 10 min.
Final temperature	250ºC hold for 10 min.
**Injection**	
Injection temperature	250ºC
Split flow	7.5 ml/min.
Auxillary temperature	250ºC
**Detector**	
Type	MS
Detector interface temperature	250ºC
Ionization	EI
Scan mode	Full scan, m/z 50-700
